# Effect of vasopressin-induced chronic hyponatremia on the regulation of the middle cerebral artery of the rat

**DOI:** 10.1007/s00424-018-2141-0

**Published:** 2018-03-17

**Authors:** Marta Aleksandrowicz, Ewa Kozniewska

**Affiliations:** Department of Neurosurgery, Mossakowski Medical Research Centre, Polish Academy of Sciences, Warsaw, Poland

**Keywords:** Acidosis, Chronic hyponatremia, Isolated middle cerebral artery, NO, Vasopressin

## Abstract

Vasopressin (arginine vasopressin, AVP) plays a crucial role in maintaining body fluid homeostasis. Excessive release of vasopressin can lead to hyponatremia. Changes in cerebral circulation during vasopressin-induced chronic hyponatremia are not elucidated. The present study has been designed to investigate the effect of chronic vasopressin-induced hyponatremia on the regulation of the tone of the middle cerebral artery (MCA) of the rat. Chronic hyponatremia was induced in vivo with the help of vasopressin, released continuously from subcutaneously implanted ALZET mini-osmotic pumps, and a liquid diet. After 3.5 days of chronic hyponatremia, the plasma Na^+^ concentration decreased to 119 ± 3 mM. MCAs were isolated and placed in a MOPS-buffered saline solution containing 121 mM Na^+^. Chronic hyponatremia did not affect the response of the MCA to increased intravascular pressure, to the administration of acetylcholine (ACh) and nitric oxide (NO) donor (SNAP, S-nitroso-N-acetyl-DL-penicillamine), and to increased K^+^ concentration, but impaired the response of the MCA to increased extravascular H^+^ concentration. Disturbed response of the MCA to acidosis was associated neither with the impairment of K_ATP_ channels nor with the activation of vasopressin V_1_ receptor. Correction of hyponatremia did not restore the response of the MCA to acidosis. These results indicate that cerebral blood vessels do not fully adapt to prolonged vasopressin-induced hyponatremia.

## Introduction

Although hyposmotic hyponatremia is a relatively common clinical water-electrolyte balance disorder which may be asymptomatic, it may result in serious complications in patients with central nervous system (CNS) diseases [12, 27]. The first complication of hyponatremia is connected with osmotically driven brain edema [12] and the second complication is the dehydration of the brain as a consequence of too rapid correction of prolonged hyponatremia [29].

Hyposmotic brain edema triggers an adaptive mechanism, referred to as “regulatory volume decrease” (RVD). The initial step of this mechanism consists of the rapid extrusion of K^+^ and Cl^−^ ions, and the obligatory efflux of water which is detectable within 3–24 h of the onset of hyponatremia [21]. The second step involves the slow loss of organic solutes, such as taurine, glycine, glutamine, myo-inositol, sorbitol, and creatine [24]. The decrease in organic osmolytes occurs within 2 days of hyponatremia [36].

The brain symptoms of hyponatremia depend on how fast the decrease in plasma sodium concentration occurs. The rapid onset of the disorder (within less than 48 h), referred to as acute hyponatremia, is associated with severe brain edema and a greater risk of death. When the onset of hyponatremia is slow (more than 48 h), the mechanism of RVD allows for the effective reduction of brain edema. As a result, symptoms of slowly developing chronic hyponatremia are milder or not diagnosed [7, 26].

Previous studies on the impact of chronic hyponatremia on the CNS focused on issues related to brain edema, such as analysis of brain water and electrolyte content [7, 34] or related to the development of brain demyelination after too rapid correction of hyponatremia [29].

According to the literature, chronic hyponatremia has a negative impact on the regulation of the cerebral blood vessels, despite the fully developed adaptive mechanism of RVD. Chronic hyponatremia induced by desmopressin in rabbits has been shown to impair cerebrovascular reactivity to hypercapnia and autoregulation to hypotension [23]. Furthermore, the functional impairment of cerebral microcirculation was reported in rats after 3.5 days of hyponatremia, particularly when low sodium concentration in the plasma was associated with an increased level of plasma vasopressin [16]. Vasopressin is known to play a crucial role in the development of hyponatremia in humans [7]. It acts through V_2_ receptors located at the distal and collecting tubules of the kidney causing increased reabsorption of water [15]. Vasopressin also has profound vascular effects acting through V_1a_ receptors located in smooth muscle [15]. Recently, it has been shown that prolonged increase in vasopressin levels in plasma can lead to oxidative stress and upregulation of endothelin-1 (ET-1) in the cerebral blood vessels [11].

Therefore, we hypothesize that chronic hyponatremia associated with an increased plasma level of vasopressin has an adverse effect on the mechanisms regulating cerebral blood vessels, such as metabolic, myogenic, and endothelium-dependent regulation. To prove this hypothesis, the reactivity of pressurized MCAs, isolated from rat brains with chronic (3.5 days) vasopressin-induced hyponatremia, to increase of intravascular pressure, ACh, NO donor, extravascular increase of K^+^ and H^+^ ions, and extravascular increase of H^+^ ion concentration with K_ATP_ channels activator or specific antagonist of V_1_ vasopressin receptor in the background, was studied. In addition, the response of the MCA to acidosis was also studied 2 h after in vitro restoration of normonatremia.

## Material and methods

### Animals

Male Wistar rats (body weight 250–300 g, *n* = 65 animals) used in this study were supplied by the Animal House of the Mossakowski Medical Research Centre, Warsaw, Poland. All animal experiments were performed in accordance with the Law and Regulations on Animal Protection in Poland (Dz.U. 2015/266) and were approved by the Extramural First Committee for the Care and Use of Laboratory Animals for Experimental Procedures, National Medicines Institute in Warsaw.

### Induction of chronic hyponatremia

Chronic hyponatremia was induced in vivo within 3.5 days using a vasopressin-filled mini-osmotic ALZET pump (Model 2002, Durect Corp., Cupertino, USA) and a rodent liquid diet (AIN-76, Bio-Serv, NJ, USA), according to Verbalis [34]. First, the rats were acclimated for 2 days to a rodent liquid diet administered in the amount of 75 ml and supplying 1.0 kcal/mL. During this time, they had free access to drinking water. Osmotic pumps filled with vasopressin were implanted subcutaneously on the neck under chloral hydrate anesthesia. The pump released vasopressin at a rate of 2.4 μg/24 h. Starting from the first day following implantation of the pump, the animals were fed the liquid diet AIN-76 containing 14% glucose in the amount of 50 mL/24 h. At this time, drinking water was withheld to prevent excessive water intake with escape from antidiuresis [35]. After 3.5 days of AVP infusion, the animals were decapitated under 5% isoflurane in 70% NO_2_/30% O_2_ anesthesia and the middle cerebral arteries were isolated as described below and blood samples were collected to measure the osmolality and concentration of Na^+^ in plasma.

Control rats were housed individually for the duration of the experiment with free access to drinking water and standard rat chow.

### Isolated cerebral artery studies

After decapitation, the brains of the normonatremic control rats were removed from the cranium and placed in a cold (4 °C, pH = 7.4 adjusted by NaOH) physiological saline buffered with MOPS (3-(N-morpholino)propanesulfonic acid) (MOPS-PSS) containing 3.0 mM MOPS, 145.0 mM NaCl, 3.0 mM KCl, 2.5 mM CaCl_2_, 1.5 mM MgSO_4_, 1.21 mM NaH_2_PO_4_, 0.02 mM EDTA, 2.0 mM sodium pyruvate, 5.0 mM glucose, and 1% dialyzed bovine serum albumin (BSA). The brains of the rats with chronic hyponatremia were placed in a buffer identical in composition to the one described above, except for the NaCl concentration which was reduced to 121 mM. The osmolality of the solutions was 300 and 250 mOsm/kg H_2_O, respectively. Isolation and cannulation of the MCAs has been described in detail elsewhere [4]. In brief, after dissection from the brain, the MCAs were transferred to the organ chamber filled with cold MOPS buffer with 1% BSA, containing either 145 or 121 mM Na^+^. The vessels were cannulated and fixed on glass micropipettes. The organ chamber was placed on the stage of the inverted microscope (CKX41, Olympus, Germany) equipped with a video camera and a monitor for analysis of internal diameters of the arteries. The vessels were pressurized to 80 mmHg intravascular pressure and perfused with MOPS containing 1% BSA and 145 or 121 mM Na^+^ to maintain normonatremia or hyponatremia, respectively. Extraluminal fluid was replaced with MOPS without BSA, containing appropriate concentration of Na^+^, slowly heated to 37 °C and exchanged at a rate of 20 mL/min.

Under such conditions, the vessels were allowed to equilibrate for 1 h to develop a myogenic tone, i.e., a contraction by about 30–40% of the diameter measured directly after pressurizing. Smooth muscle cell function was tested by increasing K^+^ ion concentration from 3 to 20 mM in an extraluminal bath.

The responses of the MCAs to increasing intravascular pressure, NO-dependent ACh, and NO donor SNAP, increased concentration of H^+^ and K^+^ ions, increased H^+^ ion concentration with K_ATP_ channels activator or with antagonist of V_1_ vasopressin receptor in the background were studied in separate arteries in the control and in the chronic hyponatremia group. In addition, the response of the MCA to increased H^+^ ion concentration was tested 2 h after the correction of chronic hyponatremia (i.e., return to the solution with Na^+^ concentration of 145 mM and osmolality of 300 mOsm/kg H_2_O).

In order to study the myogenic response of the MCA (constriction of the artery in response to increasing intravascular pressure), the intraluminal pressure was slowly lowered from 80 to 20 mmHg and then slowly raised from 40 to 100 mmHg, in 20 mmHg steps. The pressure was maintained for 10 min at each step before the diameter was measured. Next, the intravascular pressure was set again at 80 mmHg and the artery was allowed to equilibrate for 15 min in a calcium-free (1 mM EGTA) MOPS-buffered saline solution. The pressure steps were repeated according to the paradigm used prior to EGTA administration. This enabled the calculation of the diameter of MCA as a percentage of maximal dilation at each pressure step according to the formula *D*_Ca_/*D*_0Ca_ × 100, where *D*_Ca_ and *D*_0Ca_ are the diameters measured in the presence and absence of Ca^2+^, respectively.

ACh was administered intraluminally in the following concentrations: 10^−6^ M, 10^−5^ M, and 10^−4^ M, and the diameter of the MCA was measured 15 min later. Only one concentration was studied in each experiment in order to avoid tachyphylaxis.

NO donor SNAP (10^−5^ M) was administered to the extravascular buffer, and the response of the MCA to this drug was assessed after 15 min.

Hyperkalemia was induced for 15 min by the increase of K^+^ ion concentration in the extravascular fluid from 3 to 20 mM.

Acidosis was induced for 15 min by adding 2.5% HCl to the extravascular buffer which resulted in a decrease of pH from 7.4 to 7.0. The effects of the activator of K_ATP_ channels (pinacidil, 10^−5^ M) or the antagonist of V_1_ vasopressin receptor ([Deamino-Pen^1^, O-Me-Tyr^2^, Arg^8^]-Vasopressin, 10^−8^ M) and increased H^+^ ion concentration with these compounds in the background were measured after 15 min of their extravascular administration.

Changes in the diameter of the MCAs in response to the tested compounds were calculated as a percentage of the diameter measured before the administration of the compound.

All reagents used in these experiments were purchased from Sigma-Aldrich.

### Plasma analyses

During decapitation, blood was collected into heparinized Eppendorf tubes. Blood samples were centrifuged within 10 min (3000×*g*). Na^+^ concentration in plasma was measured by flame photometry (Jenway PFP7, Essex, UK). The osmolality of plasma was estimated by freezing-point depression using a semi-micro osmometer (Osmomat 030, Gonotec, Germany).

### Statistics

Statistical analyses were performed using Statistica 10 software. The results are expressed as means ± S.E.M. The data were analyzed using one-way ANOVA followed by Tukey’s multiple comparison tests, or paired or unpaired Student’s *t* test, as appropriate. Differences with *p* < 0.05 were considered statistically significant.

## Results

After 3.5 days of chronic hyponatremia, the body weight of the animals did not change. Not a single animal died due to chronic hyponatremia. Plasma Na^+^ concentration of hyponatremic rats decreased to 119 ± 3 mM (*p* < 0.001, *n* = 30), and plasma osmolality decreased to 252 ± 2 mOsm/kg H_2_O (*p* < 0.05, *n* = 6).

**Fig. 1:**
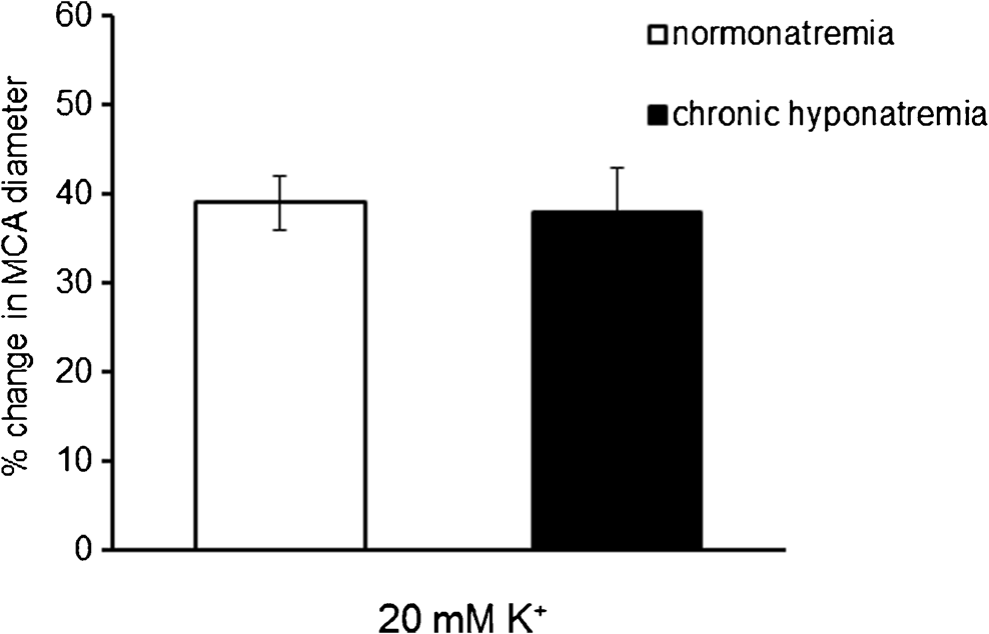
Response of the MCA to the extravascular increase in K^+^ concentration from 3 to 20 mM in normonatremia (*n* = 31) and in vasopressin-induced chronic hyponatremia (*n* = 35). MCA diameter is expressed as a percentage of baseline diameter. Data are expressed as means ± S.E.M

The diameter of the MCAs isolated from rats with chronic hyponatremia decreased from 255 ± 3 μm to 173 ± 4 μm (*p* < 0.001, *n* = 31) after 1-h equilibration on 80 mmHg intravascular pressure, in a buffer containing 121 mM Na^+^. This response was comparable to that observed in control normonatremic vessels. Extravascular increase in K^+^ concentration from 3 to 20 mM led to a comparable statistically significant dilation of the MCA in normonatremia (39 ± 3%, *p* < 0.001) and in hyponatremia (38 ± 5%, *p* < 0.001), (Fig. 1). Correction of hyponatremia (return to the buffer containing 145 mM Na^+^, 300 mOsm/kg H_2_O) caused an increase in MCA diameter by 19 ± 6%, (from 167 ± 11 μm to 197 ± 11 μm, *p* < 0.05, *n* = 5).

### Pressure-diameter relationship of MCA

**Fig. 2:**
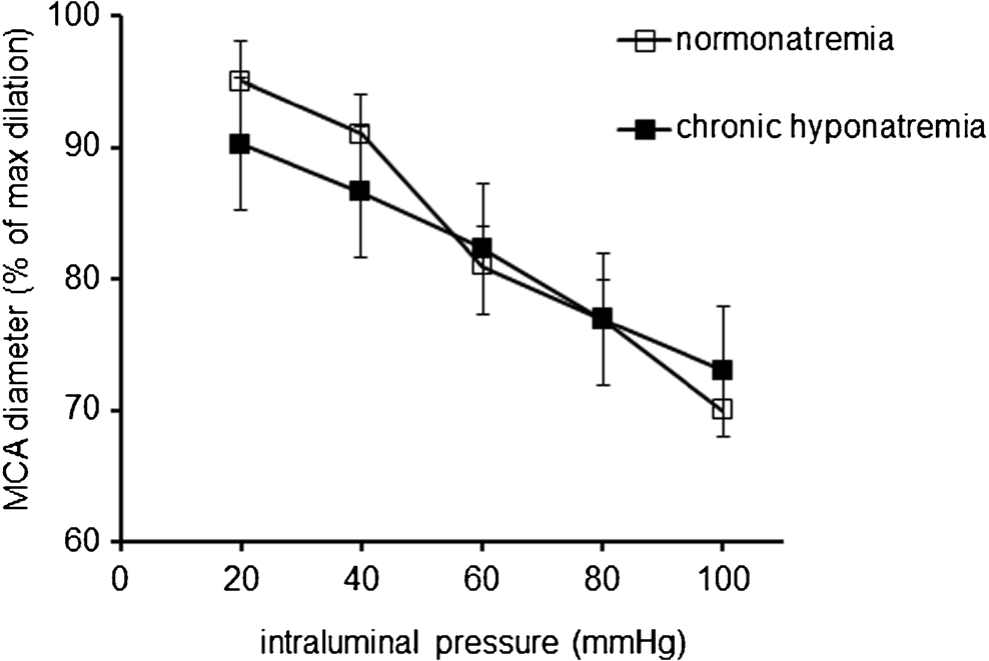
MCA pressure-diameter relationships in normonatremia (*n* = 5) and in vasopressin-induced chronic hyponatremia (*n* = 5). Diameter of the MCA is expressed as a percentage of the maximal dilation measured at each pressure in Ca^2+^-free solution (EGTA 1 mM). Data are expressed as means ± S.E.M

Increases in intravascular pressure from 40 to 100 mmHg resulted in a comparable constriction of isolated rat MCA regardless of the level of natremia. In the absence of Ca^2+^ in MOPS-PSS (1 mM EGTA), step elevations in pressure elicited increases in the diameter of the MCA in normonatremic and hyponatremic vessels. The ratio of the diameters recorded in the presence and absence of Ca^2+^ in MOPS-PSS indicates that chronic hyponatremia does not affect the myogenic response of MCA (Fig. 2).

### Response of MCA to ACh and SNAP

**Fig. 3:**
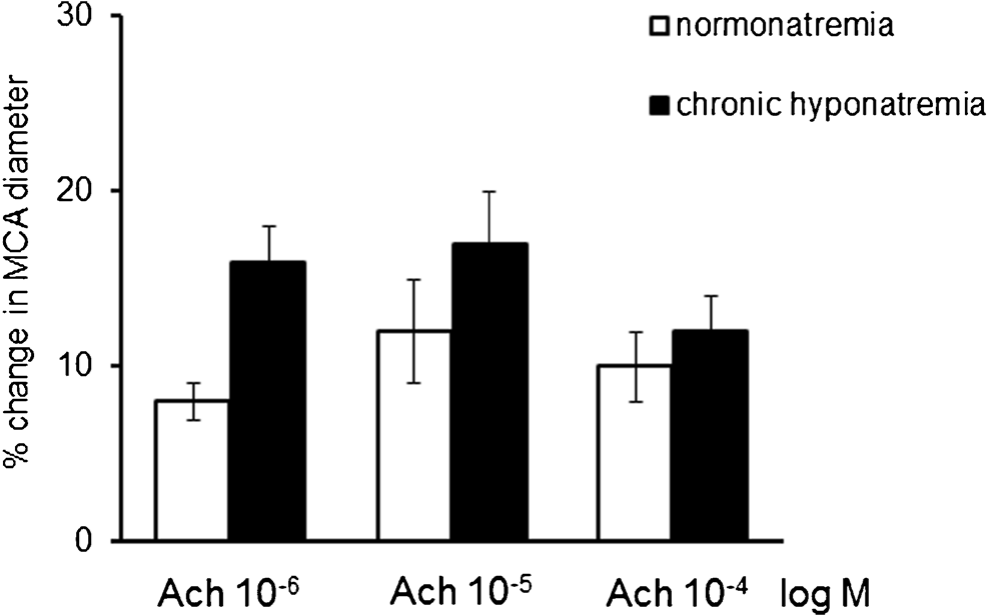
Response of the MCA to intravascular administration of endothelium-dependent vasodilator ACh (10^−6^ M, 10^−5^ M, 10^−4^ M) in normonatremia (*n* = 14) and in vasopressin-induced chronic hyponatremia (*n* = 14). Diameter of the MCA is expressed as a percentage of baseline diameter. Only one concentration was studied in each experiment. Data are expressed as means ± S.E.M

**Fig. 4:**
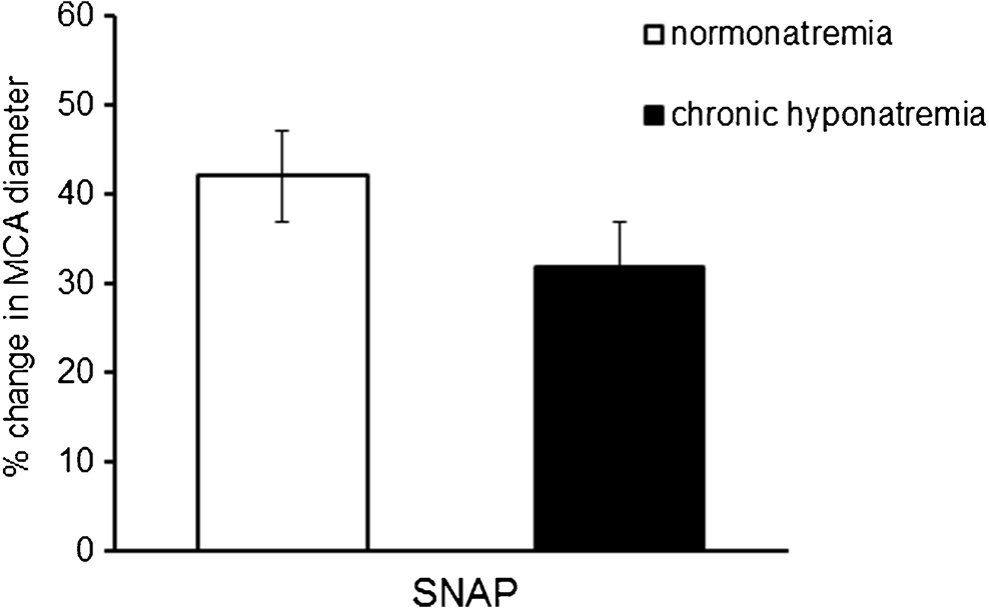
Response of the MCA to extravascular administration of NO donor SNAP (10^−5^ M) in normonatremia (*n* = 6), and in vasopressin-induced chronic hyponatremia (*n* = 6). Diameter of the MCA is expressed as a percentage of baseline diameter. Data are expressed as means ± S.E.M

Intravascular administration of endothelium-dependent vasodilator ACh (10^−6^ M, 10^−5^ M, 10^−4^ M) elicited a statistically significant and comparable relaxation of the MCA in normonatremia and in hyponatremia (Fig. 3). SNAP (10^−5^ M), an exogenous NO donor increased the MCA diameter by 45 ± 4%, *p* < 0.001 in normonatremia and by 32 ± 4%, *p* < 0.05 in hyponatremia. There were no significant differences between the groups (Fig. 4).

### Response of MCA to acidosis

**Fig. 5:**
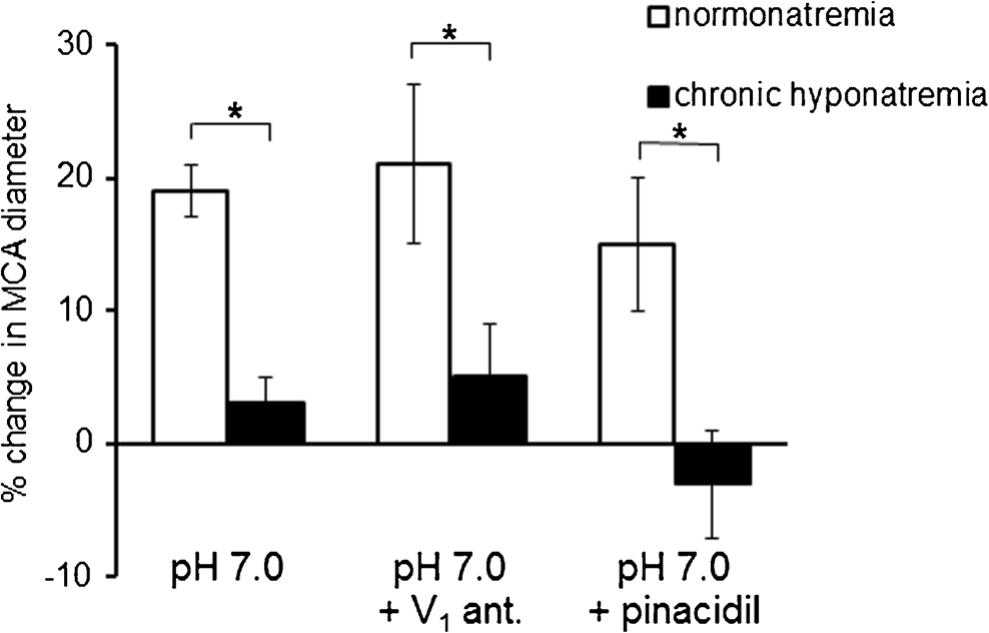
Response of the MCA to the reduction of pH from 7.4 to 7.0 in normonatremia (*n* = 14) and in vasopressin-induced chronic hyponatremia (*n* = 14). Neither the K_ATP_ channel activator, pinacidil (10^−5^ M), nor the antagonist of V_1_ vasopressin receptor, [Deamino-Pen^1^, O-Me-Tyr^2^, Arg^8^]-Vasopressin (10^−8^ M), was able to restore the response to acidosis in hyponatremia (*p < 0.05, between the groups). Diameter of the MCA is expressed as a percentage of baseline diameter. Data are expressed as means ± S.E.M

A reduction of extravascular pH from 7.4 to 7.0 elicited a statistically significant increase in the MCA diameter by 19 ± 2% (*p* < 0.001) in normonatremia. In contrast, in hyponatremia, a reduction of pH to 7.0 did not cause statistically significant dilation of MCA (3 ± 2%, NS), (Fig. 5). Correction of hyponatremia (return to the buffer with normal Na^+^ concentration and osmolality) did not improve the response of the MCA to acidosis (4 ± 2%, NS).

### Effect of K_ATP_ channels activator on the response of MCA to acidosis

Pinacidil (10^−5^ M), a specific activator of the K_ATP_ channel, led to a comparable relaxation of the MCA in normonatremia and in chronic hyponatremia, by 29 ± 6%, *p* < 0.05 and 29 ± 4%, *p* < 0.05, respectively. Presence of pinacidil did not affect the response to acidosis either in normonatremia or in hyponatremia (Fig. 5). Combined application of pinacidil and acidosis caused relaxation of MCA in normonatremia by 15 ± 6%, *p* < 0.05, which was not significantly different from the response observed without pinacidil. The deprived response of the MCA to acidosis in hyponatremia was not improved by pinacidil, indicating that the K_ATP_ channel is not impaired in chronic vasopressin-induced hyponatremia (Fig. 5).

### Effect of V_1_ vasopressin receptor antagonist on the response of MCA to acidosis

[Deamino-Pen^1^, O-Me-Tyr^2^, Arg^8^]-Vasopressin (10^−8^ M), a selective antagonist of V_1_ vasopressin receptor neither changed the basal MCA diameter nor the response of the vessel to acidosis in normonatremia. The MCA diameter increased during acidosis combined with V_1_ receptor antagonist by 21 ± 6%, *p* < 0.05, which was not significantly different from the response to acidosis observed without an antagonist. In contrast, administration of the V_1_ receptor antagonist in chronic hyponatremia resulted in a statistically significant dilation of the MCA by 19 ± 2%, *p* < 0.05. However, in an acidotic environment with V_1_ receptor antagonist in the background, there was no further vasorelaxation, i.e., blocking of V_1_ receptor did not improve the response of the MCA to acidosis in chronic hyponatremia (Fig. 5).

## Discussion

The main finding of this study is that cerebral blood vessels do not fully adapt to prolonged vasopressin-induced hyponatremia. Although autoregulation, the response to hyperkalemia, and NO-dependent dilation were well-preserved, the response to acidosis was abolished. Disturbed response of the MCA to acidosis was associated neither with the impairment of K_ATP_ channels nor with the activation of vasopressin V_1_ receptor. In this study, prolonged hyponatremia was induced according to Verbalis [34], using mini-osmotic pumps releasing vasopressin at the rate of 2.4 μg/24 h and a rodent liquid diet formula with 14% dextrose addition. Such an infusion rate of AVP produced an increase in the vasopressin concentration in the plasma of the rats to about 17 pg/mL [34], which is a concentration comparable to that observed in patients with the SIADH [37]. Under experimental conditions, chronic hyponatremia can be induced by either vasopressin or by a peptidergic analogue of vasopressin—desmopressin (dDAVP) [34]. In our study, vasopressin was chosen due to its clinical implementation in the development of hyponatremia in patients with central nervous system disorders [27]. Vasopressin is known to affect cerebral circulation in a complex manner. It has been reported to constrict small cerebral arterioles and dilate large cerebral arteries in vitro [30]. Studies performed in vivo revealed that intracarotid administration of vasopressin elevates cerebral blood flow (CBF), and this effect is associated with an increase in cerebral oxygen consumption. This vasopressin-induced increase of CBF was mediated by V_2_-like receptors but not by V_1_ receptors [17]. Recently, Faraco et al. reported that prolonged increase in vasopressin levels in plasma cause oxidative stress and upregulation of ET-1 in the cerebral blood vessels [11].

We did not observe mortality in rats with chronic hyponatremia, which is consistent with the results published by Verbalis [34]. In contrast, some studies in hyponatremia induced by the administration of vasopressin have reported mortality rates up to 30% in male rats and up to 60% in female rats [7, 13]. However, in these quoted studies, chronic hyponatremia was induced with subcutaneous AVP at higher doses and intraperitoneal 140 mM dextrose in water was given two to five times over 3 to 4 days.

Our previous studies indicate that acute hyponatremia, which was induced in vitro without the presence of vasopressin, did not affect autoregulation, but blunted the response of the MCA to ACh, NO donor, and acidosis [4, 5]. Acute hyponatremia also decreased the sensitivity of the BK_Ca_ channels in vascular smooth muscle cells to agonists [4], which would explain the impaired response of the MCA to the abovementioned vasodilators. The results of this study do not speak in favor of the impairment of the BK_Ca_ channels in the MCA during vasopressin-induced chronic hyponatremia as the response of this vessel to ACh and NO donor is unchanged. In support of this, it has been demonstrated that vasopressin had no direct effect on the K_Ca_ channels [32]. Interestingly, there are data in the literature demonstrating that vasopressin inhibits another type of K^+^ channel involved in the response of the MCA to acidosis, the K_ATP_ channel. Wakatsuki et al. have shown, using the patch-clamp technique, that vasopressin directly blocked the K_ATP_ channels in smooth muscle cells isolated from the porcine coronary artery [32]. Another study performed on smooth muscle cells isolated from the mesenteric rat artery also confirmed that vasopressin produced a concentration-dependent inhibition of pinacidil-activated currents [28]. However, in the present study, activator of K_ATP_ channels did not restore the response of the MCA to acidosis in chronic hyponatremia, indicating that disturbed response to acidosis was not associated with the decreased sensitivity of these channels to their agonists. It should be stressed, however, that our previous studies [4] as well as the studies of Lindauer et al. [18] indicated that the response of the MCA to acidosis mainly depends on BK_Ca_ channels, at least, in acute hyponatremia and in physiological conditions.

Since chronic hyponatremia was induced in our study with the help of vasopressin, we decided to test the possible impact of the activation of V_1_ vasopressin receptors to the disturbed response of the MCA to acidosis. Although vasopressin was absent in the buffer during reactivity testing of the MCAs, the vessels were exposed to this peptide for 3.5 days, which might have left a long-term imprint on them. Indeed, antagonist of V_1_ vasopressin receptor evoked dilation of the MCA in ex vivo chronic hyponatremia, while it did not change vessel diameter in ex vivo normonatremia. It is difficult to interpret the result obtained in hyponatremia, although, we see two putative explanations. The first one is that during chronic hyponatremia, there is a change in the receptor-peptide interaction resulting in a slower dissociation of vasopressin from the receptor in hyponatremic buffer without vasopressin. If this is the case, in our experimental set-up in which the extravascular buffer circulates in a closed circuit, vasopressin may appear and exert vasoconstriction acting on smooth muscle V_1_ receptors. This explanation has to be verified in a separate study. Another possibility is that the antagonist used in this experiment is a partial agonist of V_1_ receptors and activates NO synthase in the endothelium resulting in the endothelium-dependent vasodilation [30]. The latter explanation is less likely, as it has been shown by Manning et al. that [Deamino-Pen^1^, O-Me-Tyr^2^, Arg^8^]-Vasopressin has no evident vasopressor agonism, i.e., does not activate V_1_ receptor [20].

The present study also showed that combined application of an antagonist of V_1_ vasopressin receptor and acidosis did not improve the response of the MCA to a decrease of pH in chronic hyponatremia, indicating that disturbed response of the MCA to acidosis is not associated with vasopressin. Similar results were obtained in studies by Nelson et al., who noted that cerebrovascular reactivity to hypercapnic acidosis was impaired in a model of chronic hyponatremia induced not with vasoactive vasopressin but with desmopressin [23]. Thus, Nelson’s and our present results suggest that the impairment of the response of cerebral blood vessels to hypercapnic (Nelson et al.) and normocapnic (present study) acidosis in chronic hyponatremia is related to a prolonged decrease of plasma osmolality and a decreased of sodium concentration. Another study demonstrated that vasopressin does not affect the response of the MCA to hypercapnic or normocapnic acidosis in normonatremia [25]. Thus, disturbed response of the MCA did not depend on either attenuation of the K_ATP_ channels or V_1_ vasopressin receptor.

In an attempt to answer the question why the MCA does not dilate in response to extracellular normocapnic acidosis in chronic hyponatremia in our study, performed in the artificial buffer with no CO_2_ and HCO_3_^−^, one has to consider the effect of a decrease of extracellular pH (pHo) on the intracellular pH (pHi) and the participation of pHi in vasodilation during extravascular acidosis. According to Tian et al. [31] and Aalkjaer and Poston [1], decrease of pHo results in a fall of pHi in smooth muscle cells but steady-state relaxation of the MCA of the rat depends on the decrease of pHo. Moreover, according to Tian et al., decrease of pHi results in the increase of vascular wall tension in acute state, i.e., does not cause vasodilation [31]. This observation was later confirmed on smooth muscle cells isolated from the MCA of the rat by Apkon et al. [6].

It should be stressed that most of the studies on the mechanisms of acidotic vasodilation, including these cited above, were performed under physiological conditions in which the cells defend themselves against intracellular acidification by extruding H^+^ ions. There are at least two mechanisms which are activated by intracellular acidosis and are responsible for the extrusion of the protons. These are voltage-gated proton channels (Hv1) and Na^+^/H^+^ exchanger [8, 9]. Both of them were shown to be localized in vascular smooth muscles [9, 19]. The operation of Na^+^/H^+^ exchanger requires physiological transmembrane Na^+^ concentration gradient which is disturbed in hyponatremia and might result in less effective regulation of pHi in smooth muscle cells [2]. Under such conditions, one may expect greater impact of pHi than pHo on the tension of vascular wall during normocapnic acidosis. This aspect of the regulation of acidotic vasodilation in cerebral circulation during chronic hyponatremia is interesting and requires further studies.

The correction of hyponatremia in our study (i.e., return to the solution with Na^+^ concentration of 145 mM and osmolality of 300 mOsm/kg H_2_O) did not restore the response of the MCA to acidosis, at least within 2 h following the normalization of osmolality and sodium concentration. It seems that the presence of vasopressin for 3.5 days during induction of chronic hyponatremia resulted in a selective limitation of the functional adaptation to hyponatremic conditions. Another study showed that vasopressin limited the regulation of cell volume during hyponatremia [14, 33]. It should also be stressed, that at 2 h after normalization of osmolality and natremia, the cells of the MCA wall might be still in the phase of regulatory volume increase (RVI) [10]. The reversion to normoosmolality after 3.5 days of hypoosmolality is sensed by the cells as a change from normal to hyperosmolar environment which results in osmotic shrinkage and subsequent RVI.

The restoration of natremia and osmolality to physiological values in the present study caused vasodilation of the MCA. Another study showed that correction of chronic hyponatremia increased cerebral blood flow, without changes in arterial CO_2_ tension and mean arterial blood pressure [3]. Although, cerebral blood flow measured in the study by Adler’s et al. [3] represents a different level of cerebral circulation than the MCA used in our experiments, an increase in the diameter of the MCA will undoubtedly result in an increase in the flow through microcirculation in the area supplied by this artery.

In conclusion, presented results demonstrate that in chronic vasopressin-induced hyponatremia, the response of the MCA to increased extracellular H^+^ concentration is impaired despite the preservation of other important mechanisms of adjustability such as autoregulation or endothelium-dependent regulation. As acidosis is one of the main factors regulating cerebral blood flow, impaired response of the cerebral blood vessels to an increased concentration of H^+^ would have an adverse effect on neurological patients with chronic hyponatremia. This is a particularly important issue as those patients usually have symptoms of hyponatremic encephalopathy with brain acidosis [22]. Further studies need to be performed to elucidate the mechanisms behind the lack of functional adaptation of the cerebral blood vessels to chronic vasopressin-induced hyponatremia.
